# Early thrombus detection in the extracorporeal membrane oxygenation circuit by noninvasive real-time ultrasonic sensors

**DOI:** 10.1038/s41598-024-59873-z

**Published:** 2024-05-07

**Authors:** Gongmin Rim, Kwanyong Hyun, Deog Gon Cho, Zhongsoo Lim, Byungdoo Lee, Keunho Kim, Ga young Yoo

**Affiliations:** 1 Department of Thoracic and Cardiovascular Surgery, The Catholic University of Korea, St. Vincent’s Hospital, 93 Jungbu-Daero, Paldal-gu, Suwon-si, Gyeonggi-do 16247 Republic of Korea; 2 TFYHNN, Pohang, Republic of Korea; 3 Department of Thoracic and Cardiovascular Surgery, The Catholic University of Korea Seoul St. Mary’s Hospital, Seoul, Republic of Korea

**Keywords:** Extracorporeal membrane oxygenation, Real-time non-invasive thrombus detection, Thrombus, Travel time difference, Ultrasonic sensors, Biophysics, Cardiology

## Abstract

Thrombus formation in extracorporeal membrane oxygenation (ECMO) remains a major concern as it can lead to fatal outcomes. To the best of our knowledge, there is no standard non-invasive method for quantitatively measuring thrombi. This study’s purpose was to verify thrombus detection in an ECMO circuit using novel, non-invasive ultrasonic sensors in real-time, utilizing the fact that the ultrasonic velocity in a thrombus is known to be higher than that in the blood. Ultrasonic sensors with a customized chamber, an ultrasonic pulse-receiver, and a digital storage oscilloscope (DSO) were used to set up the measuring unit. The customized chamber was connected to an ECMO circuit primed with porcine blood. Thrombi formed from static porcine blood were placed in the circuit and ultrasonic signals were extracted from the oscilloscope at various ECMO flow rates of 1–4 L/min. The ultrasonic signal changes were successfully detected at each flow rate on the DSO. The ultrasonic pulse signal shifted leftward when a thrombus passed between the two ultrasonic sensors and was easily detected on the DSO screen. This novel real-time non-invasive thrombus detection method may enable the early detection of floating thrombi in the ECMO system and early management of ECMO thrombi.

## Introduction

Extracorporeal membrane oxygenation (ECMO) is an effective temporary treatment tool used in patients with severe cardiac or pulmonary diseases, as the machine supports the patient while the underlying diseases are being treated^1^. It has played a role as a last-resort treatment modality during the coronavirus disease 2019 (COVID-19) pandemic^2^. For cardiopulmonary or pulmonary support, venovenous (VV) or venoarterial (VA) cannulas are placed in the patient and connected to the ECMO circuit, which includes a membrane oxygenator and centrifugal pump. However, owing to its structure and patient factors, thrombosis remains an inevitable concern despite improvements in ECMO circuits^3,4^. These issues are related to the interaction between the synthetic circuits and blood as well as hemostatic thrombus formation around the membrane oxygenators^5^. In 2017, the Extracorporeal Life Support Organization (ELSO) reported a circuit thrombosis rate of 15–21%^6^. Thrombi within the circuit flowing to the patient can lead to fatal outcomes, such as cerebral, myocardial, renal, and other organ infarction due to embolism^7^.

Current clinical methods for detecting thrombi in an ECMO system use laboratory data (such as D-dimer and platelet count measurements), evaluation of the gas exchange capability of the oxygenator, and visual observation of the ECMO circuit by medical staff^8^. Additionally, pressure measurements of ECMO circuits are clinically used to quickly identify the thrombus^9^. However, none of the methods monitor the thrombus passing through the ECMO circuit in real-time.

Here, we introduce a novel, non-invasive method for detecting thrombi in a modified ECMO circuit in real-time, using ultrasonic sensors. Since this method cannot be attempted in human clinical practice, due to ethical concerns, we conducted in vitro experiments with the aim of verifying the efficacy.

## Materials and methods

This study aimed to detect a thrombus in a modified ECMO circuit where blood flows at a low to moderate speed (L/min [LPM]). Although it differs depending the on center’s ECMO weaning protocol, many centers apply incremental decreases in flow of 0.5 LPM to a minimum of 2 LPM, especially VA ECMO^10^. Therefore, we defined flow < 2, 2–4, and > 4 LPM as low, moderate, and high speed, respectively.

As shown by Huang CC^11^, the ultrasonic attenuation, backscattering, and velocity increase during the process of coagulation. The attenuation and backscattering are sensitive to the onset of coagulation in general but may have a drawback to be influenced by any external background noise. Our primary interest was not the onset of coagulation but the fatal introduction of macro-sized thrombi, which can be shown by the velocity measurements. Therefore, the ultrasonic speed was used as a surrogate marker to monitor clot formation along the modified ECMO circuit.

### Ultrasonic sensors with a customized chamber

**Figure 1:**
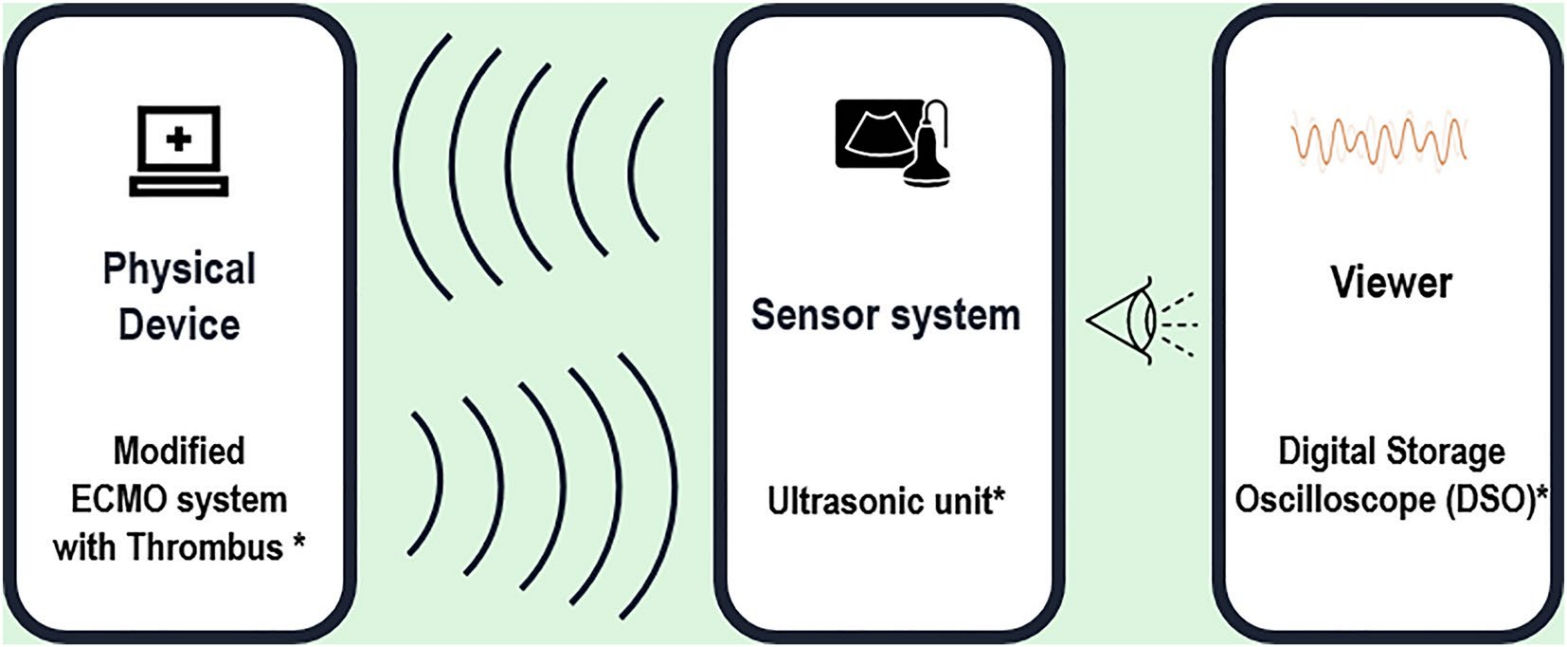
Experimental system layout for real-time thrombus monitoring. The modified ECMO system is a circuit with the membrane oxygenator removed to prevent an iatrogenic thrombus from getting stuck inside the membrane oxygenator. The ultrasonic unit included ultrasonic sensors with an ultrasonic pulser-receiver. The DSO displays ultrasonic waves at the screen.

A thrombus monitoring system was created using a modified ECMO circuit, which included a custom-built 5-MHz ultrasonic sensor with a 1/4″ diameter and 2″ focal length, a JSR DPR35 + ultrasonic pulser-receiver, and a Siglent SDS1104X-E digital storage oscilloscope (DSO). The measurement setup is shown in Fig. 1.

**Figure 2:**
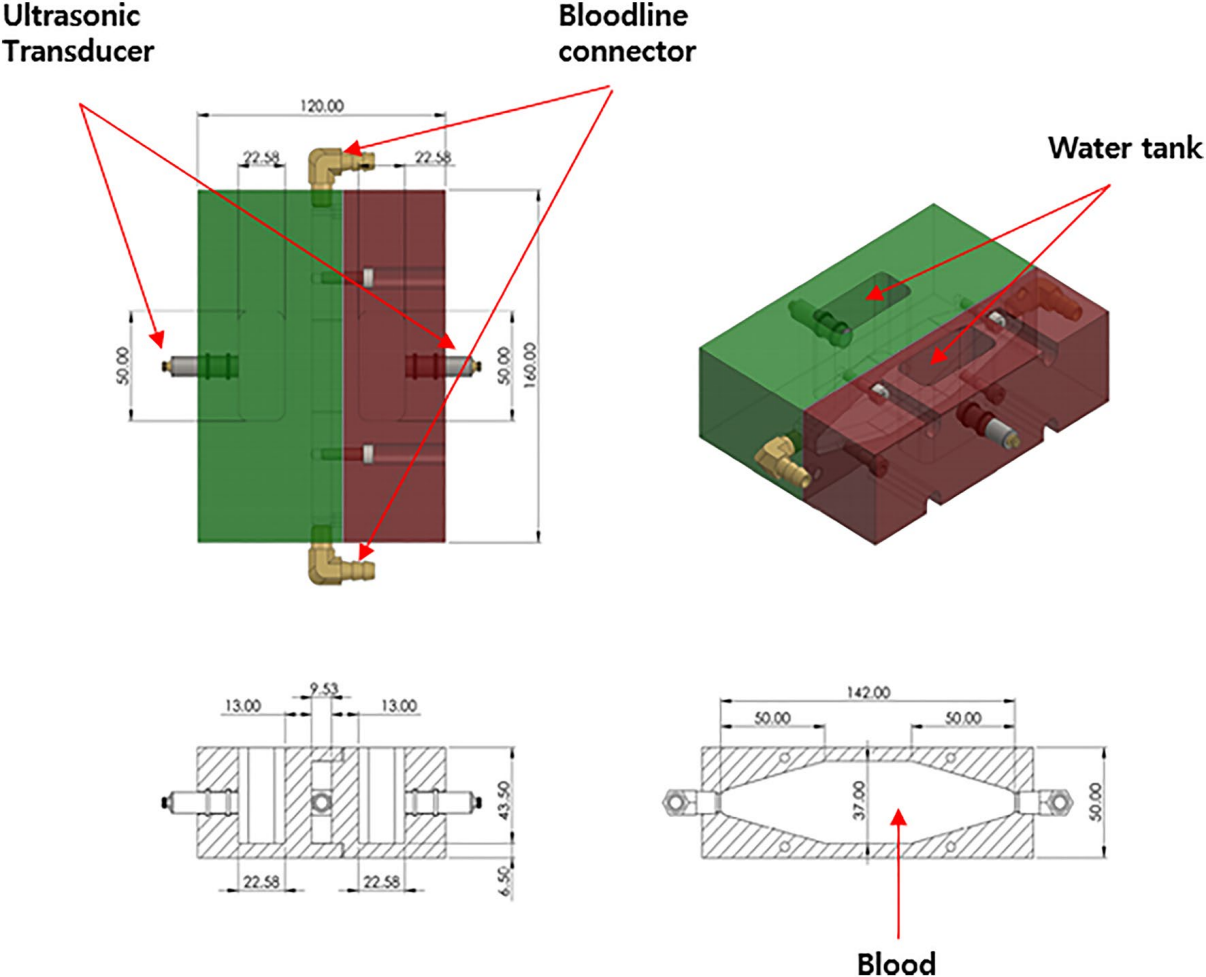
Ultrasonic chamber in the real-time thrombus monitoring system. The high-frequency ultrasonic wave cannot propagate through the air. We inserted a water tank with parallel walls to make an interpretation of the ultrasonic waveforms simpler.

The bloodline was cut, and a custom chamber was inserted to provide space to monitor the flowing thrombus using an ultrasonic pulser running perpendicular to the blood flow. To prevent multiple complicated reflections caused by the circular shape of the bloodline, the chamber had a set of parallel acryl walls. The details are shown in Fig. 2.

### Oscilloscope

A digital oscilloscope was connected to the ultrasonic pulser**-**receiver (US P/R). The US P/R had a high pulse repetition rate of more than 1000 pulses per second, enabling monitoring of the entire priming volume. The waveforms displayed on the screen were visually monitored and recorded using a digital camera in video mode.

**Figure 3:**
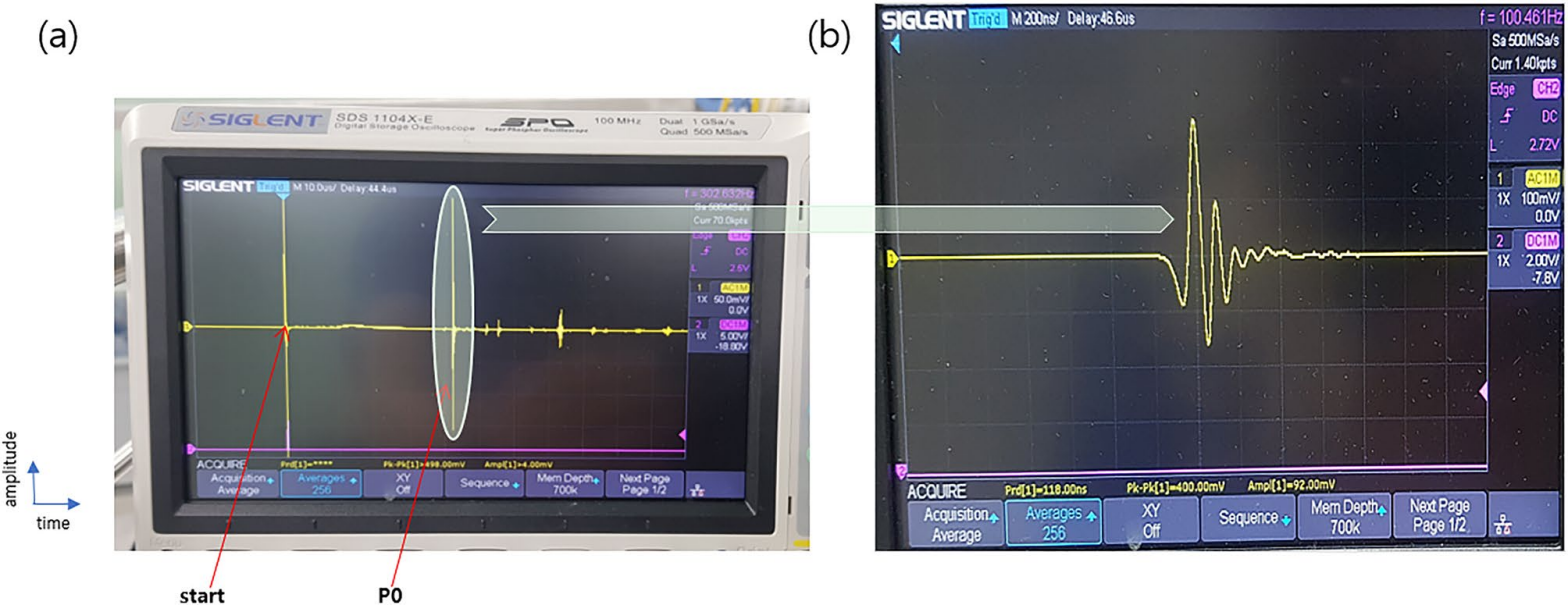
Ultrasonic waveforms at the DSO. (**A**) Full waveform showing multiple ultrasonic pulses with different paths through the ultrasonic chamber (**B**) Magnified ultrasonic pulse waveform with the lowest travel time, first arrived pulse wave (P0).

The full waveform contained many pulses from various wave paths with different arrival times, as shown in Fig. 3A. From the numerous pulses in the entire waveform, only the first arriving pulse with the lowest travel time (P0) was inspected, which revealed the travel time difference through the thrombus, as shown in Fig. 3B.

### Modified ECMO circuit

A CAPIOX EBS Circuit with X coating (Terumo Corporation, Tokyo, Japan) was used. The circuit consisted of a membrane oxygenator with microporous polymethylpentene hollow fibers, and a centrifugal pump to circulate blood through the line. However, in this study, the membrane oxygenator was removed from the circuit to prevent an iatrogenic thrombus from getting stuck inside the membrane oxygenator, thus preventing detection in the line. Hence, after the membrane was removed, the line was connected to a 3/8**″** size connector. To verify the feasibility of using ultrasonic sensors to detect thrombi, the modified ECMO circuit was primed with fresh porcine blood samples. Porcine blood was utilized instead of human blood owing to ethical issues and the relatively similar nature of porcine blood to human blood.

### ECMO system

**Figure 4:**
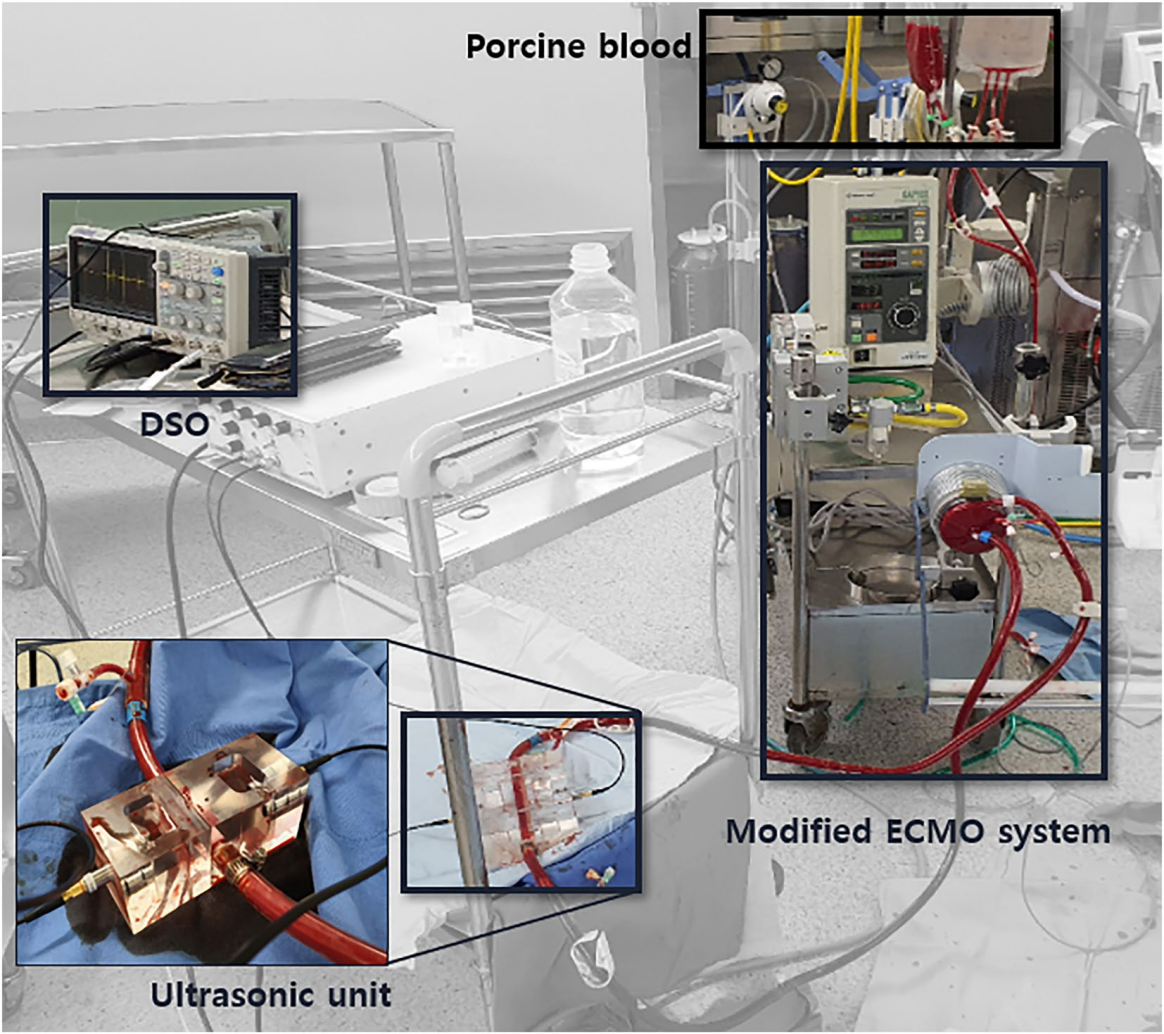
Real-time thrombus monitoring system with ultrasonic chambers. Modified ECMO system connected to ultrasonic sensors and a customized chamber. Magnified picture of the ultrasonic unit.

A CAPIOX centrifugal pump controller SP-200 ® (Terumo Corporation, Tokyo, Japan) was used in this study. The target LPM range was set to 1–4 LPM as the typical target ECMO flow rate for an adult is 60–80 mL/kg/min, and the minimum LPM for weaning from ECMO in our center was 1 LPM. Thrombus detection with ultrasonic sensors was performed using various LPMs. The modified circuit with ultrasonic sensors and the ECMO system is shown in Fig. 4.

### Thrombus

**Figure 5:**
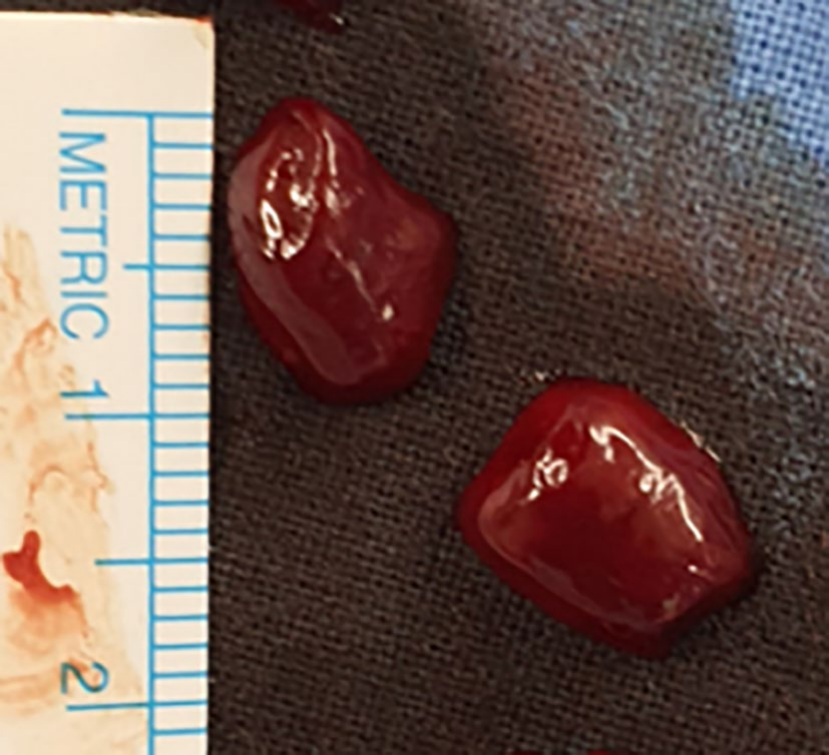
Picture of the thrombi formed from static porcine blood.

We placed three size-controlled thrombi (0.5 cm × 1 cm), formed from static porcine blood stored at room temperature for a few days, into the circuit. Figure 5 shows the shape of the thrombus.

### Porcine blood and the anticoagulation protocol

Porcine blood was obtained from a local slaughterhouse and used because of its similar characteristics to human blood, mimicking the aggregability of human blood^12^. As soon as blood was collected, a bolus dose of heparin sodium was administered for anticoagulation. In a previous study, the bolus dose of heparin used in the ECMO pig experimental model was 100 U/kg; therefore, we decided to use a bolus dose of 3000 units of heparin sodium in the porcine blood. No additional or continuous heparin was used considering the in vitro nature of the experiment. Anticoagulation was performed using size-controlled thrombus anticoagulation to prevent additional blood clot formation during the study. ACT was measured using a coagulation monitoring instrument (HEMOCHRON Signature Elite; Werfen Corp., Warrington, UK), and the activated clotting time (ACT) exceeded 200 s.

## Results

**Figure 6:**
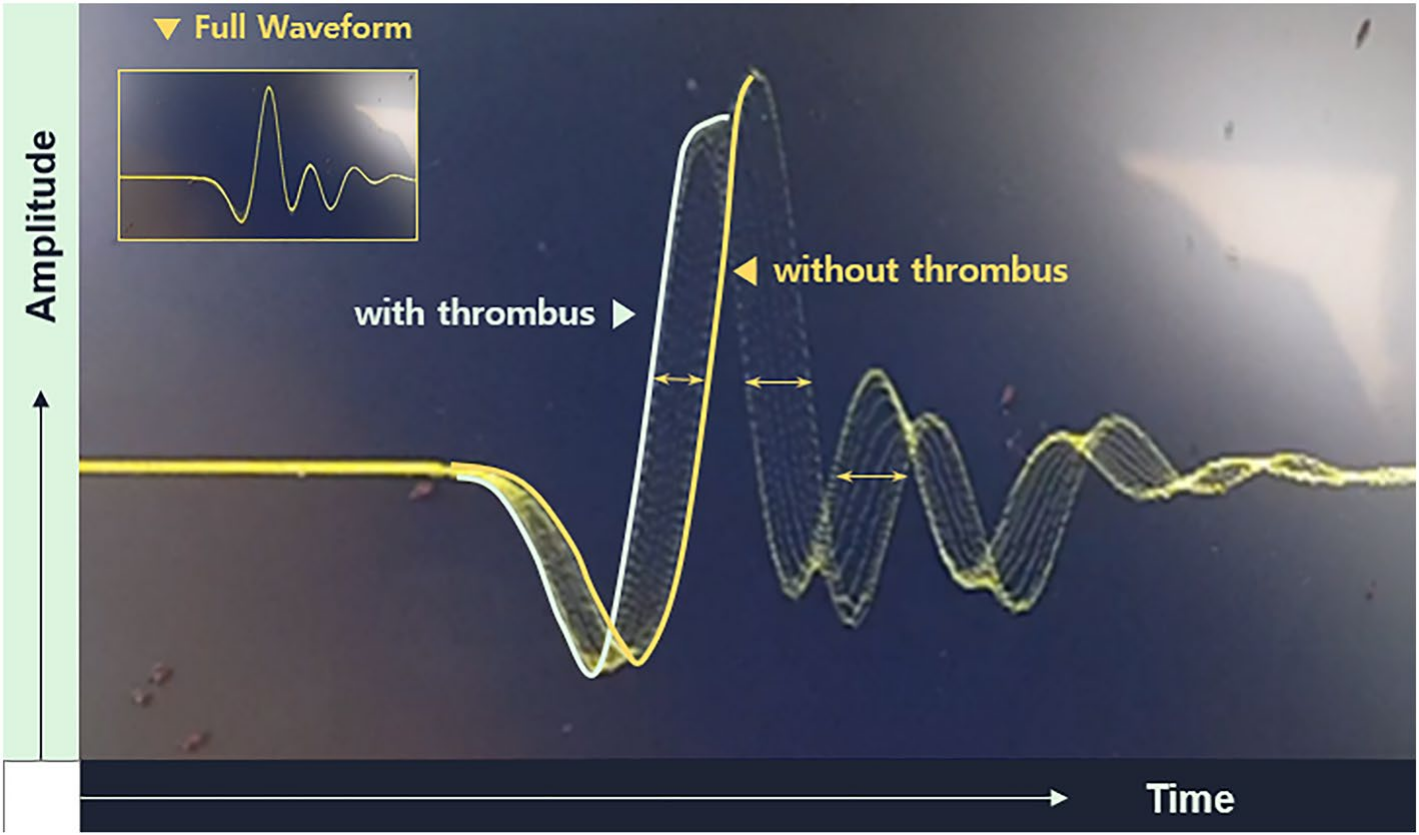
Ultrasonic signal at the DSO. Ultrasonic signal from the blood flowing in the bloodline without thrombi. The ultrasonic signal from the blood flowing through the bloodline containing a thrombus (at the flow of 1.5 LPM).

Ultrasonic signals were received from the blood flowing through the bloodline of the ECMO before and after placing the thrombi into the circuit. The ECMO priming volume was approximately 300 cc. Figure 6A shows Ultrasonic signals from the blood flowing in the bloodline without thrombi. After placing the thrombi into the bloodline, we received the ultrasonic signal shown in Fig. 6B, which shows the ultrasonic signal from the blood flowing through the bloodline containing a thrombus (at flow of 1.5 LPM).

**Figure 7:**
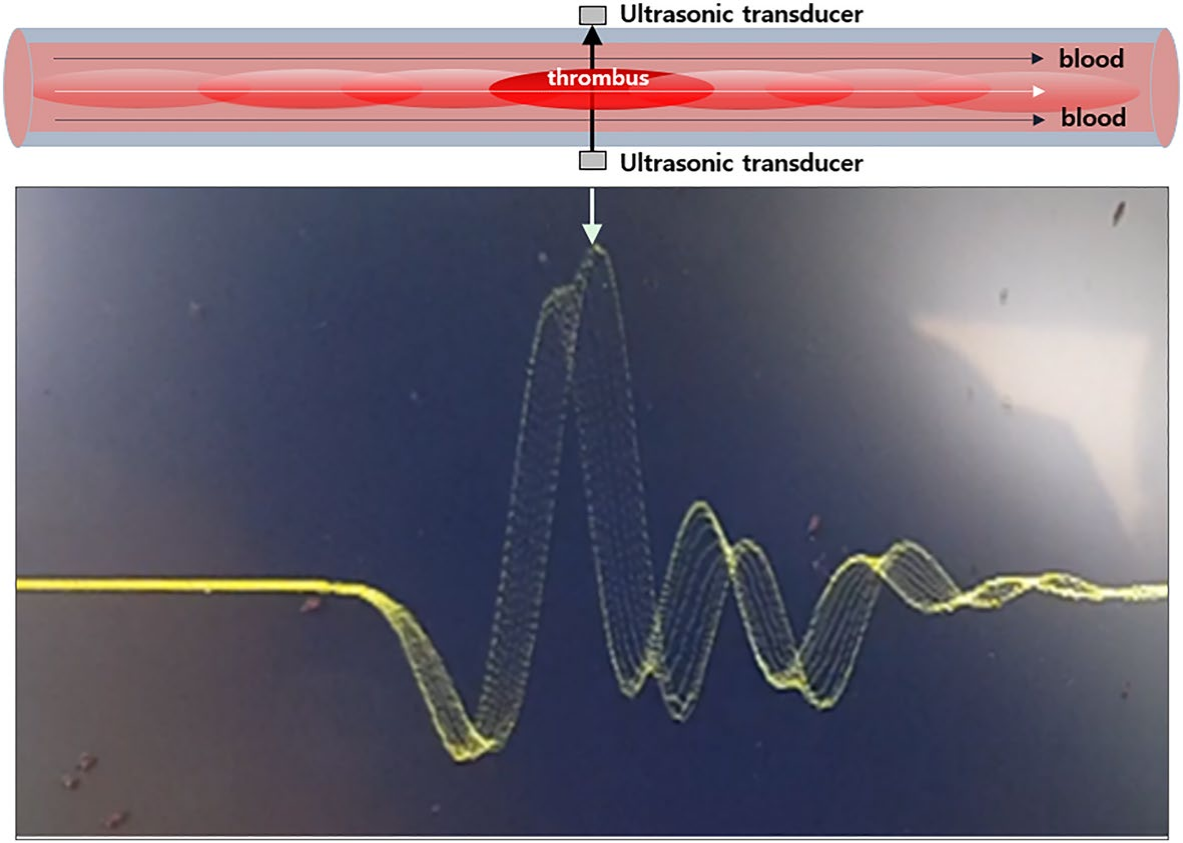
Thrombus pass through ultrasonic sensors. Thrombus flowing through the bloodline across the ultrasonic sensors. While the thrombus passes through the ultrasonic sensor, it induces multiple signal changes depending on the stages.

**Figure 8:**
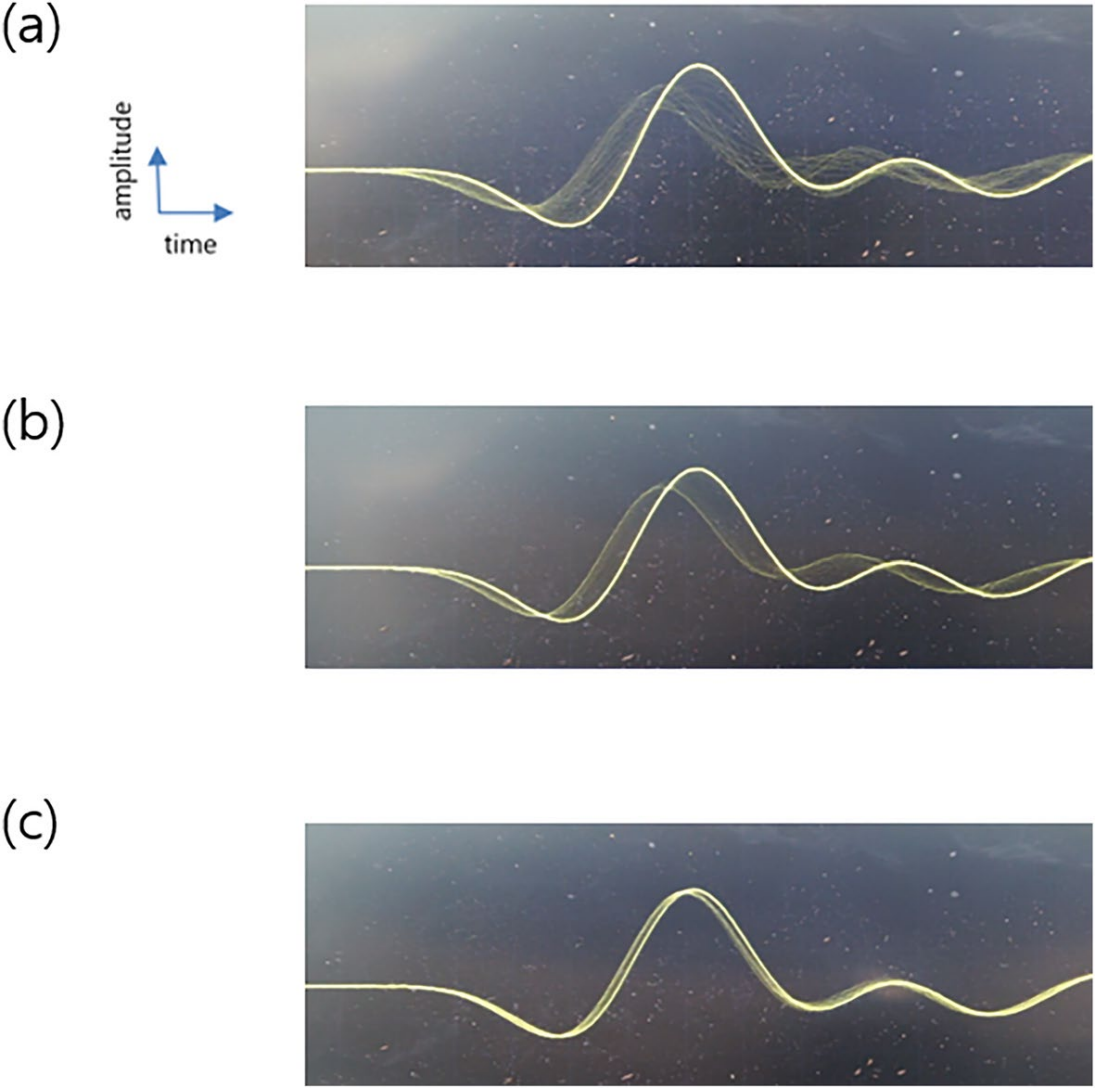
Ultrasonic signals at different LPMs. (**A**) 1 LPM, (**B**) 2 LPM, and (**C**) 4 LPM.

If the thrombus is sufficiently large, an ultrasonic signal, as shown in Fig. 7, is observed. Even one thrombus would show various stages of traversal time changes based on the relative position in the ultrasonic wave path because the ultrasonic wave would have a higher speed in the thrombus (Fig. 7). While the thrombus pass through the ultrasonic sensor, it induced multiple signal changes depending on the stage as in Fig. 7. Because we placed only a few thrombi in the bloodline, we occasionally observed transient waveform changes as the thrombus passed through the bloodline, as shown in Fig. 8.

The flow rate (up to 4 LPM) and the flowing thrombus were monitored using ultrasonic signals (Fig. 8). From Fig. 8, the size of the thrombus can be identified using the travel time difference; however, more studies are required for further analysis.

## Discussion

ECMO use continues to increase in critical care patients and has been successfully used in the management of cases of COVID-19 during the pandemic. However, thrombosis in ECMO remains frequent, often with fatal complications. Conventional ECMO thrombus detection is dependent on laboratory data such as haptoglobin, hemopexin, ACT, activated partial thromboplastin time (aPTT), anti-Xa, and evaluations of the patient’s clinical symptoms^8^. Visual observation, arterial blood gas analysis of the oxygenator membrane and pressure measurements of the ECMO circuit are other feasible methods which are clinically used methods to quickly identify the thrombus. The transmembrane pressure (TMP) is the difference between the pre- and post-membrane pressures. The TMP baseline is checked when ECMO is initiated and compared with the TMP trend during the ECMO circulation, as it reflects the function of the circuit membrane. However, it cannot determine whether the thrombus is flowing to the body through the circuit. However, despite its high incidence rate, there is no standardized and quantitative method for early, non-invasive, real-time thrombus detection^13^.

Previous studies have reported various non-invasive, real-time monitoring systems for thrombus detection based on electrical, sound, or optical principles^14–17^. However, previous studies have shown limitations such as the invasiveness of the evaluation, non-real-time evaluation, requirement for installing complex devices such as optical fibers and cameras, and measurement impracticality. Such factors are obstacles to standardized and quantitative real-time, noninvasive measurements for thrombus detection.

Ultrasonic medical techniques are widely used in medical and biological applications. Every tissue in the human body exhibits unique ultrasonic properties. However, specific tissues have different properties (velocity, attenuation, and backscattering) depending on the situation, such as blood coagulation. Shung et al. used ultrasonic backscattering to determine the onset of blood coagulation^11^. Other approaches for measuring blood coagulation, including ultrasonics, have been reviewed^18–20^.

Although previous research predominantly focused on thrombus detection within the membrane due to the majority of clotting occurrences, recent findings indicate that thrombi are also present at other sites within the ECMO circuit. For instance, a study by Figueroa Villalba et al.^21^ revealed thrombi on the surface of the inflow oxygenator membrane (11% of circuits), arterial tubing (30%), and venous tubing or connectors (26%) from the histologic, immunofluorescence, and immunohistochemical analysis. These results underscore the significance of monitoring the entire cannula line, not solely the oxygenator membrane. Furthermore, the potential consequences of thrombi traversing through the ECMO circuit to the patient can be fatal. Hence, we postulate that integrating our innovative concepts into conventional thrombus detection methods could lead to improved clinical outcome.

In this in vitro experiment, we tested the efficacy of the ultrasonic sensor at detecting floating thrombi throughout the modified ECMO circuit. By applying a 5-MHz ultrasonic sensor, ultrasonic pulser-receiver, and DSO, we could identify thrombi in the ECMO circuit at a flow rate of 1–4 LPM using the signal change of the ultrasonic wave at the DSO in real-time. As shown in Fig. 6, there were no significant signal changes with porcine blood only; however after placing the thrombus in the modified ECMO circuit, we could successfully identify the signal change when the thrombus passed through the ultrasonic sensors. As shown in Fig. 7, we hypothesized that thrombi induce traversal time changes based on their relative position in the ultrasonic wave path, as the ultrasonic wave would have a higher speed in a thrombus. We could also identify the presence of a thrombus in the tubing line from the transient waveform changes at the DSO at different LPM (1–4 LPM) as shown in Fig. 8. To the best of our knowledge, this is the first in vitro experiment to investigate the ability of ultrasonic sensors to detect thrombi within ECMO tubing lines in real-time. This preliminary study has provided us with valuable insights, indicating that ultrasonic sensors can be a useful diagnostic tool to detect thrombi in ECMO circuits. Further animal studies will be necessary to clarify these findings.

This study has some limitations. First, structural changes occurred when the oxygenator was removed from the ECMO circuit. This might have led to different physiological outcomes compared to the usual circuit, which might have resulted in different conditions for thrombi detection. Second, although the experiment was performed in an operating room that could maintain a constant atmospheric temperature, the constant temperature of the circuit could not be maintained because the membrane oxygenator was removed, which we believe was not crucial for thrombus detection. Third, because thrombus density measurements were not performed, there might be density differences in size-controlled thrombi. Because the thrombi were manually prepared, there might have been size discrepancies, despite meticulous size measurements taken during preparation. The size of the thrombi may have changed because of leakage as they flowed through the bloodline. Fourth, for ethical reasons, the circuit was primed with porcine rather than human blood, and the results might differ from those of human blood. Fifth, the detection of different-sized thrombi was not performed, and we could not determine the smallest thrombus detection size and various simulated hemodynamic statuses. Sixth, as this experiment was performed in vitro, additional experiments should be performed using a specifically developed animal model. Seventh, we believe that the current method might not be applicable for the detection of the onset of thrombus formation because, in that case, there would be too many small thrombi to be discernible by ultrasonic signals using traversal time difference measurements, and the signal change would be too small. Eighth, since the ultrasonic sensor can detect circulating thrombi or clots passing through it, the investigated method cannot detect static thrombi, such as thrombi inside the oxygenator or centrifugal pump.

## Conclusion

Using an ultrasonic measurement system, we developed a novel method for non-invasive real-time thrombus detection in an ECMO. Visual analysis of the ultrasonic signal using a digital storage oscilloscope identified the presence of a thrombus flowing through the bloodline of the ECMO. Signals from the thrombus were observed at flow rates of a maximum of 4 LPM. In addition, the size of the thrombus flowing through the bloodline could be discriminated by traversal time difference measurements; however, this requires further study, and this novel detection method should be applied in a specifically developed animal model (Supplementary Video S1).

### Supplementary Information

Supplementary Video 1.

## Data Availability

The datasets used in the current study are available from the corresponding author on reasonable request.
